# Increased Reward-Related Behaviors during Sleep and Wakefulness in Sleepwalking and Idiopathic Nightmares

**DOI:** 10.1371/journal.pone.0134504

**Published:** 2015-08-19

**Authors:** Lampros Perogamvros, Kristoffer Aberg, Marianne Gex-Fabry, Stephen Perrig, C. Robert Cloninger, Sophie Schwartz

**Affiliations:** 1 Department of Psychiatry, University Hospitals of Geneva, Geneva, Switzerland; 2 Department of Neuroscience, University of Geneva, Geneva, Switzerland; 3 Department of Psychiatry, University of Wisconsin, Madison, Wisconsin, United States of America; 4 Swiss Center for Affective Sciences, University of Geneva, Geneva, Switzerland; 5 Department of Psychiatry, Washington University School of Medicine, St. Louis, Missouri, United States of America; Oasi Research Institute, ITALY

## Abstract

**Background:**

We previously suggested that abnormal sleep behaviors, i.e., as found in parasomnias, may often be the expression of increased activity of the reward system during sleep. Because nightmares and sleepwalking predominate during REM and NREM sleep respectively, we tested here whether exploratory excitability, a waking personality trait reflecting high activity within the mesolimbic dopaminergic (ML-DA) system, may be associated with specific changes in REM and NREM sleep patterns in these two sleep disorders.

**Methods:**

Twenty-four unmedicated patients with parasomnia (12 with chronic sleepwalking and 12 with idiopathic nightmares) and no psychiatric comorbidities were studied. Each patient spent one night of sleep monitored by polysomnography. The Temperament and Character Inventory (TCI) was administered to all patients and healthy controls from the Geneva population (n = 293).

**Results:**

Sleepwalkers were more anxious than patients with idiopathic nightmares (Spielberger Trait anxiety/STAI-T), but the patient groups did not differ on any personality dimension as estimated by the TCI. Compared to controls, parasomnia patients (sleepwalkers together with patients with idiopathic nightmares) scored higher on the Novelty Seeking (NS) TCI scale and in particular on the exploratory excitability/curiosity (NS1) subscale, and lower on the Self-directedness (SD) TCI scale, suggesting a general increase in reward sensitivity and impulsivity. Furthermore, parasomnia patients tended to worry about social separation persistently, as indicated by greater anticipatory worry (HA1) and dependence on social attachment (RD3). Moreover, exploratory excitability (NS1) correlated positively with the severity of parasomnia (i.e., the frequency of self-reported occurrences of nightmares and sleepwalking), and with time spent in REM sleep in patients with nightmares.

**Conclusions:**

These results suggest that patients with parasomnia might share common waking personality traits associated to reward-related brain functions. They also provide further support to the notion that reward-seeking networks are active during human sleep.

## Introduction

Parasomnias are a category of sleep disorders involving abnormal movements, behaviors, emotions, perceptions, and dreams that may occur while falling asleep, sleeping, between sleep stages, or during arousal from sleep. Most parasomnias occur predominantly during the transitions between non-REM (NREM) sleep and wakefulness (e.g. sleepwalking, confusional arousals, sleep terrors, sleep sex, sleep driving, sleep-related eating disorder, sleep violence), or between REM sleep and wakefulness (e.g. REM sleep behavior disorder, nightmares), supporting the notion that wakefulness, NREM and REM sleep are not mutually exclusive [1]. They are often characterized by negative affect (screaming, crying) [2–4], as well as appetitive behaviors (chewing, swallowing, sleep-related eating disorder) [5] and sexual behaviors [6]. As such, the psychological and behavioral features of parasomnia episodes are suggestive of elevated activation of emotional-limbic and mesolimbic reward-seeking circuits [7].

Nightmare disorder is a parasomnia associated usually- but not exclusively- with REM sleep [8, 9]. It is found in 2–8% of the general population [8]. Nightmares often produce awakenings from sleep with recall of intensely disturbing dream content, although arousal is not a necessary condition for the diagnosis. Nightmares usually involve images, feelings or thoughts of physical aggression, interpersonal conflict, failure/helplessness, and emotions like fear or anxiety, but also anger, sadness, and disgust [3].

Sleepwalking is another frequent parasomnia. It is relatively common in childhood (3–13.5%), but its prevalence decreases during adolescence and falls to 2–4% in adulthood [10, 11]. Sleepwalking is characterized by complex behaviors that are usually initiated during arousals from slow-wave sleep and culminate in walking around in an altered state of consciousness. Sleepwalkers may show impaired judgment, as shown by difficulty in waking the person, mental confusion when awakened, complete or partial amnesia, ritualized behaviors that occur at inappropriate times, or behaviors that are inappropriate, nonsensical or dangerous [8]. Sleepwalking is associated with slow-wave sleep (i.e. N3 sleep; [11]), including frequent microarousals in N3 stage [11].

Both nightmares and sleepwalking may be idiopathic (without clinical signs of psychopathology or drug-induced) or associated with other disorders including PTSD, substance abuse, depression, stress and anxiety, borderline personality disorder, schizophrenia, and other psychiatric illnesses. Self-report epidemiologic investigations indicate that approximately 25% of adult sleepwalkers report a concurrent anxiety or mood disorder [10], although this has also been questioned [12]. Note that the association of nightmares with anxiety disorders is also inconsistent [13, 14].

The pathophysiology of nightmares and sleepwalking remains largely unknown. A genetic component has been documented for both sleepwalking [15, 16] and nightmares [17], although the functional role of this contribution is not well understood. At a neuroanatomical level, it has been proposed that nightmares involve a specific dysfunction of a network that encompasses limbic, paralimbic and prefrontal regions (e.g. amygdala, medial prefrontal cortex, hippocampus, anterior cingulate cortex), which may explain altered emotion regulation in response to stressors that are temporary (e.g., daily concerns) or persistent (e.g., trauma) [18]. By contrast, sleepwalking is a NREM parasomnia characterized by a state dissociation, in which aspects of sleep and wakefulness occur together with impaired cognitive arousal [11, 19]. The absence of NREM sleep continuity and the decrease in slow-wave activity during the first sleep cycles are both related to abnormal transient EEG activity (i.e., ‘hypersynchronous’ delta waves), which may explain the combination of features of wakefulness and sleep during sleepwalking [20, 21]. Besides, recent findings show the coexistence of sleep-like and wake-like cortical activity in nightmares too (increased high alpha power and high delta power during REM, increased low alpha power during NREM sleep and increased total alpha power during the NREM/REM transition) [22, 23]. Therefore, although sleepwalking and nightmares seem to involve different physiological characteristics, a complete dissociation between the two pathologies is unwarranted, as sleepwalking episodes are usually accompanied and may be triggered by unpleasant dreamlike mentations [2, 24].

Previous studies have only partially explored the personality traits of these patients. For example, some studies have reported increased anxiety-proneness or neuroticism in both nightmares [25–27] and sleepwalking [10, 28] compared to the general adult population, although this has been questioned [29, 30]. More research is needed to better characterize the psychological profiles of parasomnia patients and how they relate to relevant sleep features.

We recently discussed the functional importance of the activation of the mesolimbic dopaminergic (ML-DA) reward and other instinctual exploratory motivational networks (SEEKING system) [31] during NREM and REM sleep of mammals [32]. This activation would contribute to memory consolidation and the generation of dreams, and would favor the reprocessing of information with high motivational or emotional relevance [32]. Exploratory behaviors during NREM and REM parasomnias could also be related to this activation. In particular, nightmares most frequently involve a direct threat to physical integrity (e.g. being chased), with the dreamer actively searching for solutions [3]. The outcome is usually negative, resulting in the awakening of the dreamer, but can be also positive in 22% of nightmares (e.g., taking control over a situation, being finally saved) [3]. Strong positive emotions such as an unusual and exciting leisure activity can increase sleepwalking episode frequency [33], while exploratory behaviors are very frequently observed during the episode, with oriented locomotion (often modulated by dreamlike experiences, such as trying to escape imaginary threats) [24, 34]. These phenomenological characteristics of nightmares and sleepwalking suggest a disinhibition of motivated behavior during the episode.

Here we directly tested whether reward-related personality traits, such as novelty seeking, are differentially associated with primary sleepwalking and idiopathic nightmares and with specific characteristics of NREM and REM sleep. In particular, we expected that elevated activation of the ML-DA system would lead to increased reward sensitivity and constitute a common and central component in the pathophysiology of these disorders. A preliminary study in two patients with sleep-related eating disorder (SRED), a NREM sleep parasomnia characterized by recurrent episodes of involuntary eating/drinking during partial arousal from NREM sleep [35](Class IV), demonstrated that SRED patients exhibit increased reward responsiveness, experience seeking and exploratory excitability, the last three supporting increased dopaminergic neurotransmission [7]. Therefore, in the current study we hypothesized that patients with idiopathic nightmares and sleepwalking and no psychiatric comorbidity, may present elevated daytime behavioral reward seeking patterns, in particular exploratory excitability/curiosity shown to be closely related to activation in the ML-DA system [36–39]. Exploratory analyses were also performed to investigate other personality features related to anxiety-proneness and weak self-control of emotional impulses.

To test these hypotheses, we performed a detailed psychometric examination using one of the most complete inventories of personality traits, the Temperament and Character Inventory (TCI) [40]. The TCI provides a reliable quantitative way to measure configurations of emotional drives and self-regulatory functions in a way that allows consideration of both psychosocial triggers and heritable brain functions [41–43]. We also collected polysomnography data to test for any link between personality profiles and sleep parameters. In particular, it was predicted that exploratory excitability (NS1) should be related with the severity of parasomnia, as estimated by self-reported occurrences of nightmares and sleepwalking. Moreover, it was tested whether exploratory excitability is related to sleep micro- and macro structures during N3- and REM sleep for sleepwalkers and patients with idiopathic nightmares, respectively. The expected results from the present study could provide further support to the notion that reward seeking networks are active during human sleep. Overt behavior in parasomnias would then involve both motor disinhibition and elevated baseline exploratory approach, which would be uncovered by the activation of the ML-DA system during sleep.

## Methods

### Participants

#### Parasomnia patients

Twelve patients with idiopathic nightmares and twelve sleepwalkers were invited to participate in this study between February 2013 and March 2014 at the sleep clinic of the Department of Psychiatry of University Hospitals of Geneva, Switzerland. The patients sought consultation on their own or were referred by medical doctors of the Geneva area because of abnormal behaviors during the night or intense dreaming. During the first consultation, diagnosis of sleepwalking or nightmares was done by a sleep specialist and a psychiatrist according to the International Classification of Sleep Disorders (ICSD-2) diagnostic and coding manual [8]. In addition, neuropsychiatric evaluation was performed for possible comorbidity, such as depression (measured by Beck Depression Inventory/ BDI-II) [44], psychosis or anxiety disorder, including panic disorder and generalized anxiety disorder (measured by Spielberger Trait anxiety/ STAI-T) [45]. Patients with symptoms of obstructive sleep apnea syndrome, restless legs syndrome, or using dopaminergic agents or medications that would be likely to produce parasomnias (e.g. zolpidem, antipsychotic, antidepressant, anticholinergic, adrenergic drugs), patients with moderate or severe depression (Beck Depression Inventory Score >17), patients with generalized anxiety disorder (STAI-T > 57), PTSD, known psychotic disorder, as well as neurological diseases preventing the patient from completing the questionnaires due to cognitive or motor problems, or epileptic patients were all excluded. Overall, 5 patients with secondary sleepwalking (2 with generalized anxiety disorder, 2 under zolpidem treatment and 1 with schizophrenia) and 3 patients with nightmares secondary to depression (Beck Depression Inventory Score >17), were excluded. None of the subjects included in the study had symptoms of an eating disorder in their medical history. A polysomnography was then programmed two weeks after the first consultation. At the end of the first consultation, all patients first provided written informed consent and then completed the TCI, version 9 [43] (see next section), and were asked to fill in a sleep and dream diary during the next two weeks. Two weeks later, each patient came to the laboratory for the programmed polysomnography.

All twenty-four patients who were initially invited to participate in the study successfully completed the self-report questionnaires and had a polysomnography in our laboratory. The protocol was carried out in accordance with the latest version of the Declaration of Helsinki and was approved by the Ethical Committee of the Geneva University Hospitals.

#### Control participants

TCI data from 293 control participants without any psychiatric disorder were obtained (195 men, 98 women; mean age (±SD): 45 years ±12.7). These participants were recruited between 1994 and 2010 from the Blood Donor Centre of the University Hospitals of Geneva [46]. DSM-IV psychiatric diagnoses were assessed using a French version of the Diagnostic Interview for Genetic Studies [47] or the Mini International Neuropsychiatric Interview [47, 48]. All participants provided written consent and then completed the TCI.

### Personality assessment

In order to assess reward functioning and other motivational aspects of personality and behavior (e.g. approach, avoidance), we used the TCI (version 9, [43]), an inventory of 240 questions, answered true or false, that examines four temperament traits (novelty seeking, harm avoidance, persistence, reward dependence) and three character traits (self-transcendence, self-directedness, cooperativeness). Novelty seeking (NS) is a tendency to initiate appetitive approach in response to novelty and reward signals. Novelty seekers are exploratory, impulsive, extravagant, and disorderly [40, 43]. Harm avoidance (HA) involves the inhibition of behavior in response to signals of punishment or loss of reward. Harm avoidant individuals are pessimistic worriers, fearful, shy, and fatigable [40, 43]. Reward dependence (RD) is the tendency to maintain a behavior in response to cues of social reward. Reward dependent individuals are sentimental, sociable, seek social approval, and form warm attachments [40, 43]. Persistence (P) involves reward-seeking behavior despite frustration. Persistent individuals are described as hardworking, ambitious, and determined to complete tasks despite frustration and fatigue [40, 43]. Self-directedness (SD) expresses the individual’s sense of resourcefulness, purpose, and responsibility, and those who are low in Self-directedness are often described as having weak self-control [43]. Cooperativeness (CO) is characterized by being tolerant, helpful, empathic, principled, and compassionate. Self-transcendent (ST) individuals are characterized by being idealistic, easily absorbed in the moment, contemplative, and spiritual [43]. Self-transcendence involves a capacity to derive pleasure from valued actions by identifying with what is greater than one’s individual self. The temperament traits are known to correlate with specific neurobiological underpinnings [40].

### Polysomnography

Polysomnography (DeltaMed Coherence PSG, Natus, France) was recorded using 20 EEG leads according to the international 10–20 system (Fp1, Fp2, F3, F4, F7, F8, C3, C4, P3, P4, O1, O2, T3, T4, T5, T6, Fz, Cz, Pz, Ref). Right and left electrooculogram (EOG), chin electromyogram (EMG) and electrocardiogram (ECG) were recorded using conventional bipolar leads. To control for the presence of apnea and hypopnea, nasal and oral airflows were recorded with a pressure transducer (Protech2, Minneapolis, MN, USA) and thoracic and abdominal respiratory movements were acquired with strain gauges. Oxygen saturation (SaO2) was continuously measured with a finger oximeter. Right and left anterior tibialis muscle EMG activity was monitored using bipolar surface electrodes in order to study lower limb motor activity. EEG and EMG signals were sampled at 512 Hz. Sleep was scored according to new criteria using 30 s epochs [49]. We computed the total sleep time and time percentage for each sleep stage (time passed in this stage divided by total sleep time). Microarousals were scored according to the American Sleep Disorder Association’s criteria and the total number of microarousals was determined. Periodic limb movements (PLMs) with the number of PLMs per hour of sleep (periodic leg movement index, PLMI) were calculated. A PLMI greater than 15 throughout the night sleep was considered pathological. An abnormal breathing event during sleep was defined as apnea (a complete cessation of airflow lasting 10 seconds or more) or hypopnea (a reduction of more than 50% in respiratory airflow accompanied by a decrease of at least 3% in SaO2 or micro-arousal). An index of apnea-hypopnea episodes per hour of sleep (apnea hypopnea index, AHI) greater than 5 was chosen as a criterion for sleep-disordered breathing. All patients were continuously videotaped with an infrared camera during the night.

### Parasomnia severity

In the current study, patients consulted for their parasomnia because of impairment of psychosocial functioning. All sleepwalkers had more than one episode per month for at least 3 months, whereas all subjects with nightmares had more than one nightmare per week for at least 6 months prior to the consultation. Accordingly, in this study, nightmare severity was estimated according to the number of nightmares per week during the past 6 months. On the other hand, severity of sleepwalking episodes was assessed according to the number of sleepwalking episodes per month during the past 3 months. The frequency of self-reported occurrences of nightmares or sleepwalking during this time was then calculated for each patient and a total parasomnia severity score was calculated as the sum of the z-scored nightmare and sleepwalking frequencies. Z-scores were used to reduce the influence of overall difference in reported nightmare and sleepwalking frequencies.

### Statistical analysis

Instead of using traditional tests that estimate p-values from theoretical sampling distributions, p-values were obtained by comparing observed statistics with empirical sampling distributions obtained by Monte Carlo (MC) methods under the null hypothesis [50]. As an example, when performing a MC test equivalent to a two-sample *t*-test, the null distribution is constructed by i) randomly shuffling observations between the two groups, ii) recording the mean difference between the two groups, and iii) repeating this procedure a large number of times (n = 10'000 in the present study). P-values are obtained by calculating the proportion of trials in which the mean differences making up the null distribution are larger than the mean difference in the observed sample. The MC method is less sensitive to violations of normality making it more suitable for small sample size, as compared to the traditional *t*-test.

Two-sample MC permutation tests were used to compare means and categorical variables (i.e. gender differences) between groups. For comparing observed samples with a reference population with mean value μ and standard deviation σ (n participants), MC permutation tests were performed using n samples drawn from a normal distribution with mean μ and standard deviation σ in each repetition. The relationship between two variables was tested using the Spearman’s rank correlation coefficient which is less sensitive to outliers than the Pearson's correlation coefficient. Significance levels were obtained using MC methods by randomly shuffling observations for one variable and holding the other variable constant in each repetition.

All a priori directional hypotheses were tested using one-tailed MC permutation tests with significance level at 0.05. Exploratory tests were conducted using two-tailed MC permutation tests with Bonferroni corrections for multiple tests where applicable (e.g. significance level α = .05/31 = .0016 when testing the 31 TCI scales and subscales).

## Results

### Demographic and clinical characteristics

Twenty-four patients were included in the study (8 men, 16 women; mean age (±SD): 37 years ±11.8; see Table 1). The sleepwalkers and the idiopathic nightmare groups did not significantly differ with respect to age and gender (4 men, 8 women in each group). Patient groups did not differ for depression, which was within the normal range, but levels of anxiety were significantly higher in sleepwalkers compared to patients with nightmares (STAI-T mean ± SD: nightmares = 36.4 ± 6.5; sleepwalkers = 46.2 ± 5.6; p = .001). Moreover, as compared to norms for healthy subjects (i.e. mean STAI-T ± SD: 35.5 ± 9.4 for working adults between 19–39 and 40–49 as reported in [51]), sleepwalkers displayed significantly higher anxiety (p = .00008), while patients with nightmares did not (p = .72).

**Table 1:** Demographic, clinical, and sleep characteristics of patients with sleepwalking and idiopathic nightmares.

	Idiopathic nightmares (n = 12)	Sleepwalkers (n = 12)
**Demographic**		
Females (n)	8	8
Age (years)	33.4 ± 9.1	41.1 ± 13.5
**Clinical**		
STAI-T score	36.4 ± 6.5	46.2 ± 5.6 **
BDI-II score	8.0 ± 4.5	7.0 ± 4.5
**Sleep**		
N2%	54.1 ± 4.4	52.8 ± 7.2
N3%	15.1 ± 5.5	18.1 ± 6.8
REM %	22.1 ± 3.7	21.6 ± 7.4
N1 microarousals	12.1 ± 10.4	6.8 ± 6.2
N2 microarousals	27.7 ± 32.5	34.1 ± 27.7
N3 microarousals	2.4 ± 3.2	9.3 ± 13.1 *
REM microarousals	33.3 ± 22.7	4.5 ± 4.5 ***
Total microarousals	80.8 ± 59.5	49.3 ± 35.9
PLMI	5.3 ± 7.5	3.2 ± 5.3
AHI	5.4 ± 4.1	3.0 ± 2.4

Abbreviations: AHI: the index of apnea-hypopnea episodes per hour of sleep; BDI-II: Beck Depression Inventory; Microarousals: total number of microarousals; PLMI: the index of periodic limb movements per hour of sleep; N2%: percentage of N2 sleep; N3%: percentage of N3 sleep; REM%: percentage of REM sleep; STAI-T: Spielberger Trait Anxiety

* p < .05

** p < .01

*** p < .001

### Sleep results

#### Sleep macro structure

The repartition of N2, N3 and REM sleep stages was roughly similar to the one observed in healthy controls [52] (Table 1). Subjects with nightmares did not spend more time in REM sleep (mean ± SD: nightmares = 22.1 ± 3.7; sleepwalkers = 21.6 ± 7.4, p = .85), nor did sleepwalkers spend more time in N3 sleep (mean ± SD: nightmares = 15.1 ± 5.5; sleepwalkers = 18.1 ± 6.8, p = .25).

#### Sleep micro structure

The total numbers of microarousals were within normal ranges [53]. As predicted, sleepwalkers displayed significantly more microarousals during N3 sleep (mean ± SD: nightmares = 2.4 ± 3.2; sleepwalkers = 9.3 ± 13.1, p = .033 (one-tailed)), while nightmares displayed more microarousals during REM sleep (mean ± SD: nightmares = 33.3 ± 22.7; sleepwalkers = 4.5 ± 4.5, p = .00003 (one-tailed)).

#### PLMI and AHI

PLMI did not differ between patient groups (PLMI mean ± SD: nightmares = 5.3 ± 7.5; sleepwalkers = 3.2 ± 5.3; p = .44) nor did the number of apnea-hypopnea episodes (AHI mean ± SD: nightmares = 5.4 ± 4.1; sleepwalkers = 3.0 ± 2.4; p = .09).

### Personality measures (TCI)

There were no significant differences between the two groups for any of the 31 TCI scales and subscales, even in the absence of Bonferroni correction for multiple tests (Table 2). Thus, the two parasomnia groups were considered together for subsequent analyses. The hypothesis that parasomnia patients display increased exploratory excitability compared to the normal population (n = 293) was tested and confirmed (NS1 mean ± SD: parasomnia = 8.42 ± 2.43; controls = 5.95 ± 2.41, p < .00001 (one-tailed)). Moreover, exploratory analyses revealed that parasomnia patients also displayed significantly increased disorderliness (NS4 mean ± SD: parasomnia = 5.08 ± 2.45; controls = 3.64 ± 1.88, p = .0005), overall novelty seeking (NS mean ± SD: parasomnia = 23.58 ± 7.16; controls = 18.85 ± 5.56, p = .0001), anticipatory worry (HA1 mean ± SD: parasomnia = 5.00 ± 2.81; controls = 3.49 ± 2.16, p = .0013), attachment (RD3 mean ± SD: parasomnia = 5.83 ± 1.69; controls = 4.38 ± 2.11; p = .0011), and decreased self-acceptance (SD4 mean ± SD: parasomnia = 6.13 ± 2.79; controls = 8.71 ± 2.16; p < .00001) and overall decreased Self-Directedness (SD mean ± SD; parasomnia = 29.92 ± 7.71; controls = 34.31 ± 5.76; p = .0006). These data are presented in Table 3.

**Table 2:** Personality scores on the TCI for sleepwalkers and patients with idiopathic nightmares.

TCI scales/subscales	Idiopathic nightmares (n = 12)	Sleepwalkers (n = 12)
**Novelty seeking (NS total)**	24.33 ± 7.19	22.83 ± 7.37
Exploratory excitability (NS1)	8.33 ± 2.46	8.50 ± 2.51
Impulsiveness (NS2)	5.42 ± 2.64	4.50 ± 2.71
Extravagance (NS3)	5.42 ± 2.02	4.83 ± 2.13
Disorderliness (NS4)	5.17 ± 2.41	5.00 ± 2.59
**Harm avoidance (HA total)**	14.25 ± 9.89	14.92 ± 9.41
Anticipatory worry (HA1)	4.83 ± 2.92	5.17 ± 2.82
Fear of uncertainty (HA2)	3.00 ± 2.45	3.42 ± 2.19
Shyness (HA3)	3.17 ± 2.95	3.08 ± 2.64
Fatigability (HA4)	3.25 ± 2.63	3.25 ± 2.96
**Reward dependence (RD total)**	15.75 ± 4.12	16.58 ± 3.83
Sentimentality (RD1)	7.08 ± 1.83	7.42 ± 2.11
Attachment (RD3)	5.92 ± 1.38	5.75 ± 2.01
Dependence (RD4)	2.75 ± 1.91	3.42 ± 1.38
**Persistence (P)**	5.33 ± 1.61	6.17 ± 1.80
**Self-directedness (SD total)**	31.17 ± 6.01	28.67 ± 9.21
Responsibility (SD1)	6.41 ± 1.62	5.08 ± 2.94
Purposeful (SD2)	5.92 ± 1.38	5.25 ± 2.56
Resourcefulness (SD3)	4.08 ± 0.79	3.83 ± 1.59
Self-acceptance (SD4)	6.25 ± 3.11	6.00 ± 2.56
Enlightened second nature (SD5)	8.75 ± 2.30	8.50 ± 2.24
**Cooperativeness (CO total)**	32.92 ± 4.98	30.75 ± 7.38
Social acceptance (CO1)	6.92 ± 1.17	6.67 ± 1.72
Empathy (CO2)	5.67 ± 1.07	4.92 ± 1.38
Helpfulness (CO3)	6.00 ± 1.35	5.83 ± 1.64
Compassion (CO4)	7.58 ± 2.71	6.92 ± 2.97
Pure-hearted conscience (CO5)	6.75 ± 1.66	6.42 ± 1.56
**Self-Transcendence (ST total)**	15.83 ± 6.34	12.75 ± 4.94
Self-forgetful (ST1)	6.17 ± 3.04	6.42 ± 2.58
Transpersonal identification (ST2)	4.25 ± 2.01	3.50 ± 2.28
Spiritual acceptance (ST3)	5.42 ± 3.85	2.83 ± 1.95

Note: The groups did not display any significant difference at the uncorrected α = 0.05.

**Table 3:** Personality differences on the TCI between controls and parasomnia patients (sleepwalkers together with patients with idiopathic nightmares).

TCI scales/subscales	Parasomnia patients (n = 24)	Controls (n = 293)	p_uncorrected_
**Novelty seeking (NS total)**	23.58 ± 7.16	18.85 ± 5.56	0.0001 *
Exploratory excitability (NS1)	8.42 ± 2.43	5.95 ± 2.41	< 0.0001 *
Impulsiveness (NS2)	4.96 ± 2.66	4.54 ± 2.30	0.40
Extravagance (NS3)	5.13 ± 2.05	4.71 ± 1.86	0.30
Disorderliness (NS4)	5.08 ± 2.45	3.64 ± 1.88	0.0005 *
**Harm avoidance (HA total)**	14.58 ± 9.45	12.97 ± 6.18	0.24
Anticipatory worry (HA1)	5.00 ± 2.81	3.49 ± 2.16	0.0016 *
Fear of uncertainty (HA2)	3.21 ± 2.28	3.76 ± 1.89	0.18
Shyness (HA3)	3.13 ± 2.74	3.39 ± 2.28	0.59
Fatigability (HA4)	3.25 ± 2.74	2.33 ± 1.95	0.032
**Reward dependence (RD total)**	16.17 ± 3.91	14.46 ± 3.64	0.029
Sentimentality (RD1)	7.25 ± 1.94	6.83 ± 1.93	0.30
Attachment (RD3)	5.83 ± 1.69	4.38 ± 2.11	0.0012 *
Dependence (RD4)	3.08 ± 1.67	3.25 ± 1.45	0.59
**Persistence (P)**	5.75 ± 1.73	4.61 ± 1.98	0.0066
**Self-directedness (SD total)**	29.92 ± 7.71	34.31 ± 5.76	0.0006 *
Responsibility (SD1)	5.75 ± 2.42	6.57 ± 1.66	0.028
Purposeful (SD2)	5.58 ± 2.04	6.03 ± 1.72	0.23
Resourcefulness (SD3)	3.96 ± 1.23	3.98 ± 1.21	0.94
Self-acceptance (SD4)	6.13 ± 2.79	8.71 ± 2.16	< 0.0001 *
Enlightened second nature (SD5)	8.63 ± 2.22	9.02 ± 2.13	0.39
**Cooperativeness (CO total)**	31.83 ±6.25	33.46 ± 4.67	0.11
Social acceptance (CO1)	6.79 ± 1.44	6.96 ± 1.36	0.55
Empathy (CO2)	5.29 ± 1.27	5.23 ± 1.42	0.84
Helpfulness (CO3)	5.91 ± 1.47	6.17 ± 1.16	0.31
Compassion (CO4)	7.25 ± 2.80	8.35 ± 1.94	0.011
Pure-hearted conscience (CO5)	6.58 ± 1.59	6.74 ± 1.27	0.57
**Self-Transcendence (ST total)**	14.29 ± 5.78	13.93 ± 6.33	0.79
Self-forgetful (ST1)	6.29 ± 2.76	4.67 ± 2.49	0.0027
Transpersonal identification (ST2)	3.88 ± 2.13	3.80 ± 2.18	0.87
Spiritual acceptance (ST3)	4.13 ± 3.26	5.47 ± 3.19	0.049

Note: * Significant difference after Bonferroni correction for multiple comparisons (α = 0.05/31 = .0016).

### Correlations between TCI and sleep parameters

#### TCI and parasomnia severity

In our sample, 9 patients with nightmares had more than 1 episode per week but less than 1 episode per night, whereas 3 patients had more than 1 episode per night. All sleepwalkers had more than 1 episode per month but less than 1 episode per night. It was hypothesized that the severity of parasomnia should be associated with exploratory excitability (NS1). Indeed, participants reporting more frequent occurrences of nightmares and sleepwalking scored higher in exploratory excitability (NS1) (ρ = 0.36, p = .038 (one-tailed)). Exploratory analyses testing relationships between parasomnia severity and all other TCI scores using a Bonferroni corrected significance level revealed no other significant correlations (see Table 4).

**Table 4:** Correlations between parasomnia severity and TCI scores in parasomnia patients (sleepwalkers together with patients with idiopathic nightmares, n = 24).

Parasomnia severity vs.	*p*(rho)	p
**Novelty seeking (NS total)**	0.10	0.64
Exploratory excitability (NS1)	0.36	0.04*
Impulsiveness (NS2)	-0.14	0.53
Extravagance (NS3)	0.02	0.94
Disorderliness (NS4)	-0.17	0.44
**Harm avoidance (HA total)**	0.07	0.75
Anticipatory worry (HA1)	-0.11	0.61
Fear of uncertainty (HA2)	0.09	0.68
Shyness (HA3)	0.07	0.74
Fatigability (HA4)	0.14	0.51
**Reward dependence (RD total)**	0.11	0.62
Sentimentality (RD1)	-0.02	0.93
Attachment (RD3)	-0.07	0.73
Dependence (RD4)	0.26	0.23
**Persistence (P)**	0.19	0.38
**Self-directedness (SD total)**	0.19	0.36
Responsibility (SD1)	0.09	0.67
Purposeful (SD2)	0.25	0.25
Resourcefulness (SD3)	0.08	0.70
Self-acceptance (SD4)	0.17	0.43
Enlightened second nature (SD5)	0.11	0.62
**Cooperativeness (CO total)**	0.01	0.97
Social acceptance (CO1)	0.16	0.46
Empathy (CO2)	-0.26	0.23
Helpfulness (CO3)	0.07	0.75
Compassion (CO4)	-0.02	0.94
Pure-hearted conscience (CO5)	-0.001	0.99
**Self-Transcendence (ST total)**	0.08	0.72
Self-forgetful (ST1)	0.24	0.27
Transpersonal identification (ST2)	0.07	0.74
Spiritual acceptance (ST3)	-0.02	0.94

Note: Besides the significant correlation between parasomnia severity and exploratory excitability (NS1), which was tested using a one-tailed test due to a directional a priori hypothesis, no other correlations were significant, as tested using two-tailed tests, even at an uncorrected α = 0.05.

* p < .05.

#### TCI and sleep structure

Because nightmares and sleepwalking predominate during REM and NREM respectively, we tested whether increased exploratory excitability (NS1) may be associated with REM sleep parameters in the nightmare group and N3 sleep in sleepwalkers. As predicted, NS1 correlated positively with REM% sleep for nightmares (ρ = 0.67, p = 0.023), but not for sleepwalkers (ρ = 0.34, p = 0.28). However, NS1 did not correlate with %N3 sleep, neither for the nightmare group (ρ = 0.18, p = 0.59) nor for sleepwalkers (ρ = 0.34, p = .28). Moreover, while groups differed with respect to microarousals, NS1 did not correlate with the number of microarousals during REM sleep within the nightmare group (ρ = 0.14, p = 0.64) (nor within the sleepwalking group; ρ = -0.29, p = 0.36). NS1 did also not correlate with the number of microarousals during N3 sleep in either patient group (nightmares: ρ = -0.04, p = .88; sleepwalkers ρ = 0.05, p = 0.88).

## Discussion

In this study, we performed a detailed psychometric examination and whole-night polysomnography in patients with sleepwalking and idiopathic nightmares in order to test the hypothesis that elevated daytime behavioral reward patterns, as measured by specific personality traits, particularly Novelty Seeking, characterize these patients. One main result is that both sleepwalkers and patients with nightmares were high on the Novelty Seeking (NS) and low on the Self-Directedness (SD) scales of the TCI, suggesting elevated impulsivity as found in impulsive personality disorder (e.g. cluster B in DSM-IV and V). In addition, both groups of patients had high scores on the attachment (RD3) subscale, suggesting a strong reliance on social attachments. In terms of psychosocial motivation, this pattern of results suggests that parasomnia patients are likely to act impulsively to seek gratification, especially social attachments, and may have difficulty self-regulating their craving for social supports. These findings are supported by previous studies showing increased impulsivity [54, 55], emotional sensitivity [56, 57], dissociative experiences [58] and low mindfulness [59] in parasomnias.

In neurobiological terms, these findings substantiate a link between specific parasomnia behaviors occurring during sleep and waking personality traits pertaining to reward-seeking brain functions. Indeed, NS1, which was increased in both our parasomnia groups, reflects high activity within the mesolimbic dopaminergic (ML-DA) system [36–39]. In addition, the dopamine D4 receptor polymorphism (DRD4) may play a role in the partial determination of NS [60]. Here, we also found that NS1 correlated significantly with the severity of parasomnia symptoms. Thus, elevated daytime novelty seeking and impulsivity suggests increased ML-DA activity, which may extend into sleep where it would contribute to overt behaviors in sleepwalkers and increased motivational biases in nightmares.

Indeed, from a phenomenological perspective, most patients with sleepwalking report that their somnambulistic behaviors are being motivated by an intrinsic sense of urgency [11], they are related to an unpleasant dreamlike mentation [2], and are being executed with increased perseverance. Sleepwalking seems to involve increased activity in motor cortices and the cingulate cortex [19, 61, 62], which may be responsible for generating anticipatory biases toward cues and locations with elevated motivational value for the subject and moving towards them [63]. As for nightmare sufferers, they frequently report that they engage in avoidance and exploratory behaviors [3, 64], including the active search for solutions in their dreams [3], and that they experience strong emotions, like fear [18]. The convergence of the phenomenological characteristics of both sleepwalking and nightmares episodes (emotionality and motivated behaviors) with daytime reward-related personality traits (e.g., increased novelty seeking) further indicates a continuity between brain activity/behavior during wake and sleep states [65], although some significant discontinuities between dream content and waking concepts cannot be neglected [66, 67]. Therefore, along with several other lines of evidence from human and animal research [68–70], this study supports that the ML-DA reward and other motivational networks regulating reward-seeking are functionally active during human sleep [32].

In addition to reward-related traits, patients with parasomnias had higher scores on the anticipatory worry (HA1) subscale, a measure specific for pessimism and worrying about what might go wrong in future, and on the social attachment (RD3) subscale. The combination of these two subscales may lead to a hypersensitivity to loss of social supports [71], which is likely to be chronic or frequently recurrent when Self-directedness is low [42, 72], as it is in parasomnia patients. This finding is in accordance with recent studies showing that people with anxious attachment style demonstrate greater self-negativity and aggressive content in their dreams [73] and greater occurrence of sleepwalking episodes [74]. While the level of anxiety in the sleepwalkers of our study did not meet the criteria for generalized anxiety disorder, as expected given the infrequency of severe psychopathology in other studies [12, 30], both trait anxiety (STAI-T) and anticipatory worrying (HA1 subscale of TCI) measures suggest that sleepwalkers experience more frequent anxiety and worry than controls, as expected from prior research [10, 28]. It is possible that sleepwalkers could be worried and anxious due to their sleep disorder affecting their sleep and daytime functioning, so we cannot rule out the possibility that the sleep disorder is the cause of their increased anxiety and worry. However, because the measures of both STAI-T and TCI HA1 are moderately stable personal characteristics throughout life, it seems more likely that they pre-exist sleepwalking in our adult patient population. This could be tested by future prospective studies or by studies of trait anxiety in the siblings of parasomnia patients and controls, as has been done to demonstrate the role of personality traits in susceptibility to mental disorders [75, 76]. By contrast, patients with nightmares had lower trait anxiety than sleepwalkers according to the Spielberger trait anxiety measure (Table 1, 36 vs 46, p<0.01). Patients with nightmares did not differ significantly from the sleepwalkers on any TCI personality traits, but the two parasomnia groups were significantly higher in anticipatory worrying (HA1) than the controls (Table 3, 5.0 vs 3.5, p<0.01). Therefore it is likely that nightmares are associated with states of stress-related worrying, as has been observed in a study showing that the association between neuroticism and nightmares is mediated by state (and not trait) anxiety [77]. Indeed, stress increases the frequency of negative emotions in dreams and the frequency of nightmares [27]. Thus, both parasomnia subgroups share some general anticipatory fear, which is intermittently exacerbated by social rejection.

Although the repartition of sleep stages was not significantly different between groups or compared to normative data, and the total number of microarousals was not different when comparing separately each type of parasomnia with the norms [53] (contrary to the findings of [78] for sleepwalkers), there were differences between groups for the N3 microarousals (increased in sleepwalking) and REM microarousals (increased in nightmares). These observations would be consistent with nightmares being a REM disorder and sleepwalking a NREM disorder. Correlations of TCI subscales with sleep stages demonstrated that REM% positively correlated with exploratory excitability (NS1) in patients with idiopathic nightmares. Because NS1 is specifically related to ML-DA activity, a positive correlation between NS1 and REM% may support a link between dopamine, REM sleep, and intense dreaming [32].

A recent study of Park et al. [79] found that daytime personality traits may correlate with the severity of insomnia (negative correlations with novelty seeking, reward dependence, cooperativeness and positive correlations with harm avoidance, self-transcendence), suggesting a link between individual daytime reward profile and sleep. In addition, insomnia patients have increased harm avoidance and decreased self-directedness compared to controls [80]. Contrasting with these findings in insomnia, we found that parasomnia patients displayed a positive correlation between severity of their sleep disorder and exploratory excitability, but also displayed increased anticipatory worry and decreased self-directedness. Thus, our study further demonstrates the potential role of daytime personality profile in sleep disorders and, to the best of our knowledge, it is the first to explore the links between specific personality traits in sleepwalking and idiopathic nightmares, and sleep parameters in the patients.

However, our study has some limitations. Firstly, the sample size (12 patients in each subgroup) was relatively small, so that statistical power to detect differences between subgroups was limited. Considering the two subsamples together in some analyses is a second limitation, as similarities and differences with respect to personality traits would deserve further investigation in large representative samples of the two syndromes. Thirdly, the cross-sectional design of the present study precludes any definite conclusion with respect to a possible causal relationship between personality traits and parasomnias. Thus, while the patients were clearly characterized by specific personality traits, we do not claim that these traits pathophysiologically cause parasomnias, but rather that they may influence some symptomatological facets of parasomnia patients, such as the content of nightmares and specific somnambulistic behaviors. Future prospective studies with larger patient populations could help further confirm these findings. Fourth, several variables might have acted as confounders but were not assessed in the present study, such as perceived stress, coping and everyday experiences possibly influencing sleep behavior. Moreover, investigating other types of parasomnias characterized by similar motivational attributes (e.g. SRED, sleep-talking, sleep-driving, REM behavior disorder, sleep-related sexual behaviors) could provide further support to the hypothesis of the activation of reward networks and function during sleep in humans. Finally, long-term home video monitoring would certainly be useful [81], because complex and disinhibited behaviors occur more frequently in familiar environments such as at home, and a more detailed phenomenological characterization of these two disorders is certainly needed.
